# An Untypical Case of Gouty Infiltration of Both Peroneal Tendons and a Longitudinal Lesion of the Peroneus Brevis Tendon Mimicking Synovial Sarcoma

**DOI:** 10.1155/2018/8790916

**Published:** 2018-07-26

**Authors:** Konstantinos Anagnostakos, Andreas Thiery, Christof Meyer, Octavian Tapos

**Affiliations:** Zentrum für Orthopädie und Unfallchirurgie, Städtisches Klinikum Saarbrücken, Saarbrücken, Germany

## Abstract

We present a case of a 70-year-old male patient with an untypical gout infiltration of the peroneal tendons mimicking synovial sarcoma. The patient had a negative history of gout at initial presentation in our department. Magnetic resonance imaging of the region revealed a finding highly suspicious for synovial sarcoma of the peroneal tendons. Open biopsy was performed. Histopathological examination of the tissue samples demonstrated the presence of gout with no signs of malignancy. The gout infiltration was excised in a subsequent surgery. Orthopedic surgeons should be aware of the potential manifestation of gout in tendons and bear this in mind in the differential diagnosis of soft tissue tumors.

## 1. Introduction

Gout is a systemic inflammatory disease caused by deposition of monosodium urate crystals within synovial or periarticular tissue. The initial manifestation of gout is usually an acute attack of synovitis affecting a single joint, most commonly the first metatarsophalangeal joint [1, 2]. Depending on the treatment and the compliance of the patient, gout may become oligo- or polyarticular and lead to joint damage and erosive arthropathy [1, 2]. Soft tissues may also be affected, when subcutaneous nodular deposits of urate crystals (tophi) emerge, especially at the toes, Achilles tendons, prepatellar tendons, and fingers [1, 2].

In the present report, we would like to demonstrate an untypical case of gouty infiltration of the peroneal tendons mimicking synovial sarcoma in a patient with no prior history of gout.

## 2. Case Report

A 70-year-old male patient presented in our department with a painful swelling over the left lateral malleolus, which rapidly emerged over the past 3 months. The patient had no history of trauma. His medical history was completely negative regarding any comorbidities, especially no rheumatic disease, psoriasis, or gout.

**Figure 1:**
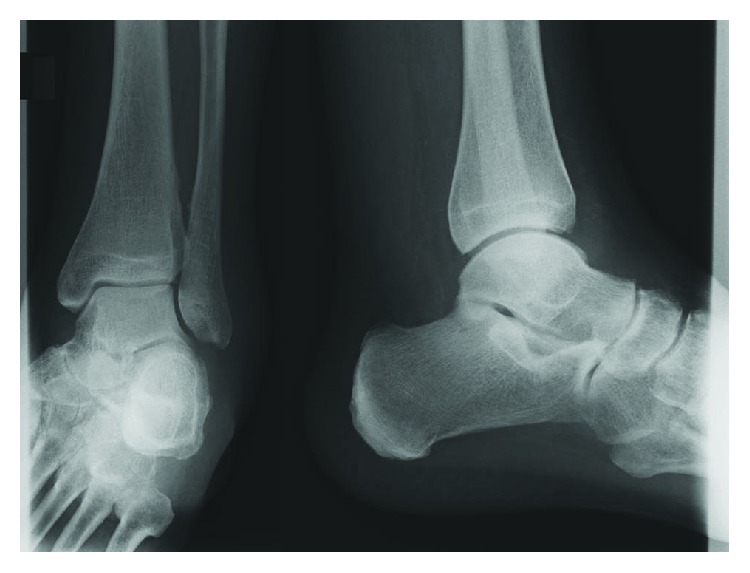
Preoperative anterior-posterior and lateral radiographs of the left ankle joint showing no signs of any osseous destruction or joint damage.

At initial presentation, a large swelling over the left lateral malleolus was evident with no redness. A local tenderness was present over the peroneal tendons but not the lateral malleolus itself. The range of motion of the left ankle joint was extension/flexion 5/0/30°. Anterioposterior and lateral X-rays of the ankle joint did not show any signs of osteoarthritis or other osseous destruction (Figure 1). For further differential diagnosis, a magnetic resonance imaging (MRI) tomography was planned.

**Figure 2:**
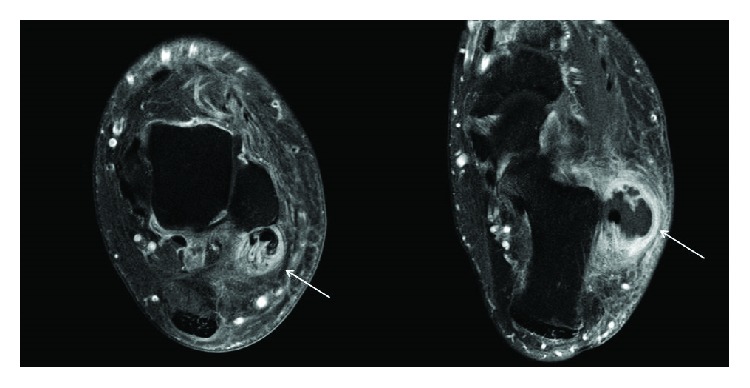
T2-weighted axial magnetic resonance images demonstrating a soft tissue tumor along and around the peroneal tendons with hypo- and hyperintense central areals and an inhomogenous, diffuse uptake of the contrast agent (arrows), highly suspicious for a synovial sarcoma.

MRI revealed a lesion of size of 4.3 × 2.7 × 3.7 cm in close relation to the peroneal tendons with hypo- and hyperintense central areals, especially in the inframalleolar area, and an inhomogenous, diffuse uptake of the contrast agent, highly suspicious for a synovial sarcoma (Figure 2). C-reactive protein concentration was 5 mg/dl, and the white blood cell count was 8800 × 10^6^/l. Based on these findings and under the suspicion of a malignant soft tissue tumor, an open incisional biopsy of the region was carried out. Tissue samples of the biopsy were sent for microbiological and histopathological examination.

The microbiological examination showed no bacteria growth. The histopathological examination revealed a chronic granulomatous inflammation with debris and deposition of urate crystals indicating tophaceous gout, with no signs of any malignancy.

The histopathological report was then discussed with the patient. At that point, gout was unknown for him and he has never had any gout-specific complaints prior to the onset of these symptoms. Laboratory examination showed a serum uric acid concentration of 7.0 mg/dl (normal values: 3.4–7.0 mg/dl).

**Figure 3:**
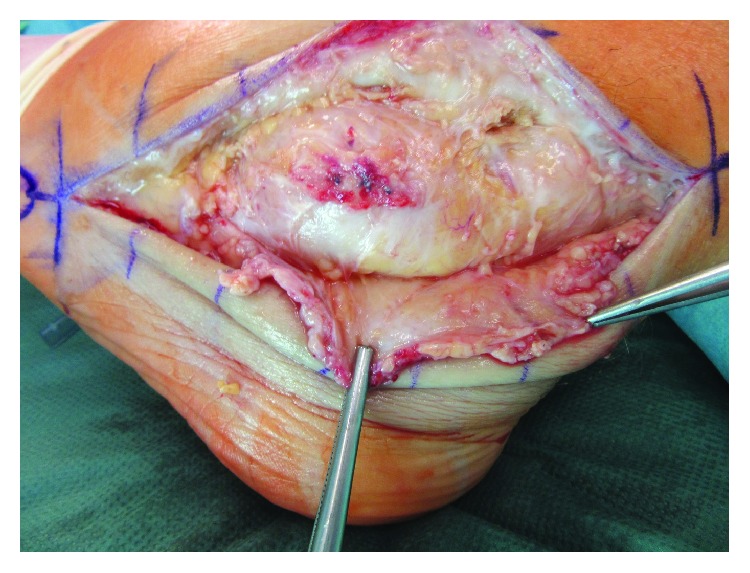
Exposure of the tophaceous lesion after opening of the tendinous sheath of the peroneal tendons.

**Figure 4:**
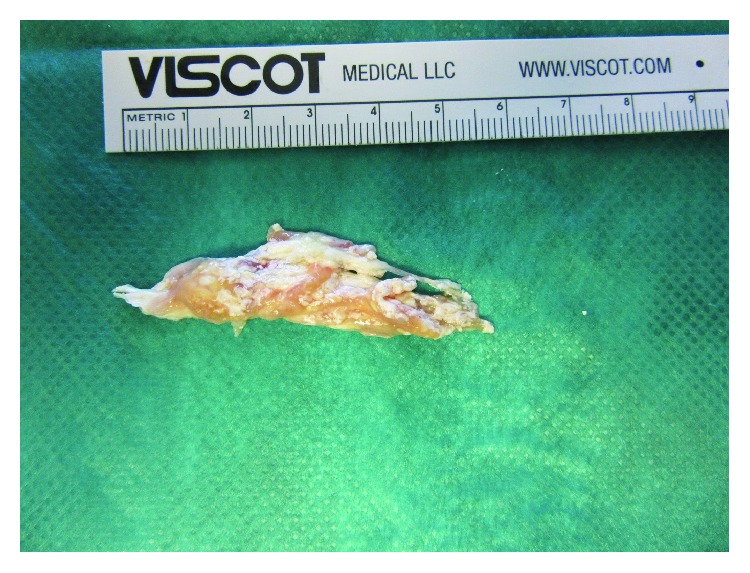
The excised tophus.

**Figure 5:**
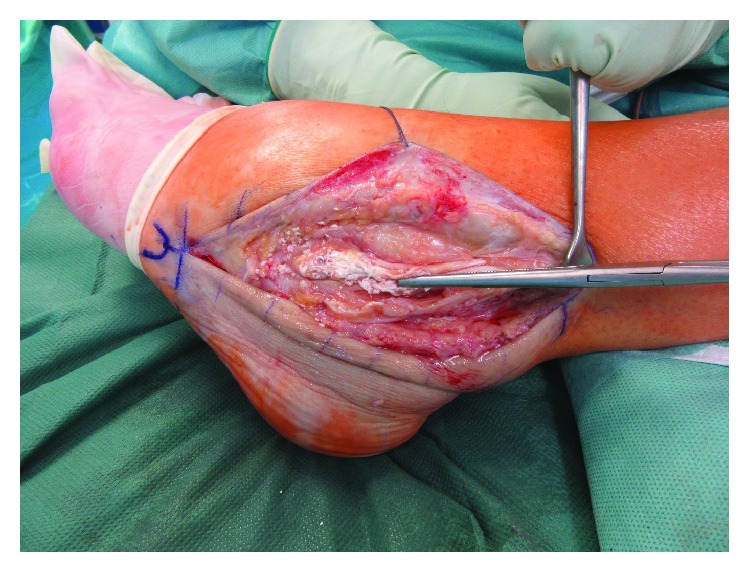
Following excision of the tophus, the gout infiltration of the tendons was evident.

**Figure 6:**
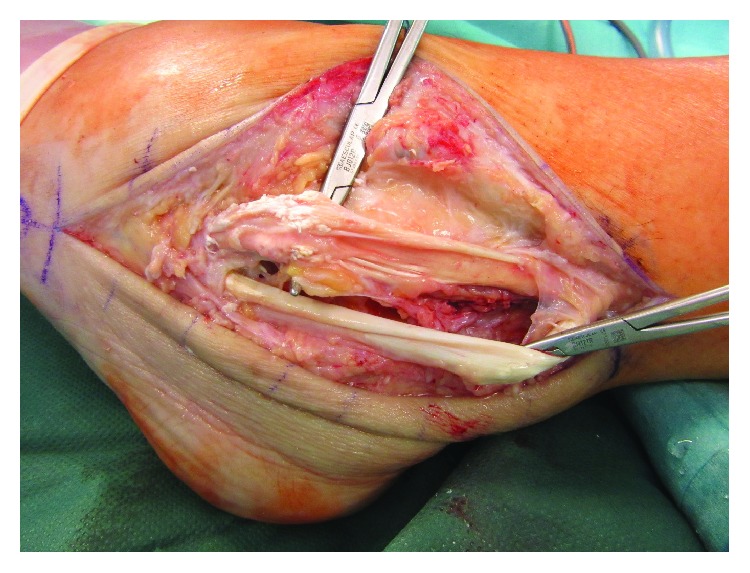
The tendons of the peroneus brevis and longus were prepared along their entire course and debrided from all tophaceous infiltration.

Due to the mechanical obstruction of the swelling, especially for shoes wearing, the patient wished for excision of the tophus. 14 days after the open biopsy, revision surgery was performed. The peroneal tendons were prepared along their entire course from the musculotendinous transition to the insertion of the peroneus brevis tendon to the fifth metatarsal bone. After opening of the tendinous sheath of both peroneal tendons, the tophaceous lesion was exposed (Figure 3). The lesion was then excised from proximal to distal (Figure 4). Both tendons of the peroneus brevis and longus were then prepared along their entire course and debrided from any remaining tophaceous infiltration (Figures 5 and 6).

Postoperatively, the operated leg was immobilized in a Vacoped® shoe (Fa. Omed, Germany) for the first 6 weeks. The patient was allowed to put weight bearing as tolerated. The further postoperative course was uneventful, and the patient was dismissed 4 days after surgery. The patient was advised to follow gout nutrition therapy along with allopurinol medication.

## 3. Discussion

The present case demonstrates an unusual manifestation of gout in a tendon area which can be easily misdiagnosed as a sarcoma on MRI, especially in patients with an asymptomatic hyperuricemia prior to its manifestation in soft tissues. Since our patient had a negative history regarding gout or other rheumatic diseases, the “tumor” showed a rapid growth within 3 months, and no structural damage of the ankle joint was evident on plain radiographs; a soft tissue tumor seemed to be the most probable initial diagnosis, which cannot be easily differentiated from gout infiltration on MRI. To the best of our knowledge, such a case with this diagnostic dilemma has not been described yet.

High levels of uric acid in biological fluids play a role in the development and progression of tendinopathy [3]. This is supported by the notion of uric acid acts as a death cell-associated stressor [3] Moreover, high concentrations of uric acid in the tendon cell microenvironment involve a mild alteration in extracellular matrix homeostasis [3]. Although gout can frequently involve tendons of the upper [4] and lower extremity [5, 6], the peroneal area is a less commonly affected one. Ventura-Rios et al. investigated the prevalence of tendon involvement in patients with gout in a multicenter, multinational study [5]. The prevalence of intratendinous aggregates and tophi in gout was most frequent at the distal patellar insertion, followed by the quadriceps, Achilles, and proximal patellar insertion ones [5]. Regarding the tendon involvement in feet, Dalbeth et al. examined the frequency of urate crystal deposition in 92 patients using dual-energy computer tomography [6]. The Achilles tendon was the most commonly involved one in 39% of the cases.

The gouty infiltration of the peroneal tendons has been scarcely reported in literature [7–9]. De Yoe et al. reported on a case with a history of chronic ankle instability and pain [7]. A MRI showed an attenuated anterior talofibular ligament and a longitudinal tear of the peroneus brevis tendon. During surgery, tophaceous gout was present within the tear of the peroneus brevis as well as the attenuated anterior talofibular ligament. Lagoutaris et al. described a case of a 35-year-old patient with longitudinal tears of both peroneal tendons associated with tophaceous gouty infiltration [8]. A similar case was also published by Radice et al. [9]. In our case, both tendons were involved by the gouty infiltration accompanied with a longitudinal lesion of the peroneus brevis tendon.

In conclusion, the gouty infiltration of tendons and soft tissues should be considered to be a rare differential diagnosis against other soft tissue tumors, even in asymptomatic patients with an unknown hyperuricemia prior to their first tophaceous manifestation. An open biopsy with histopathological examination is mandatory in these cases for exclusion of other pathologies.
